# Does the Volume and Localization of Intracerebral Hematoma Affect Short-Term Prognosis of Patients with Intracerebral Hemorrhage?

**DOI:** 10.1155/2013/327968

**Published:** 2013-05-08

**Authors:** Denisa Salihović, Dževdet Smajlović, Omer Ć. Ibrahimagić

**Affiliations:** Department of Neurology, University Clinical Centre Tuzla, Trnovac bb, 75000 Tuzla, Bosnia and Herzegovina

## Abstract

The aim of this study was to determine whether volume and localization of intracerebral hematoma affects the six-month prognosis of patients with intracerebral hemorrhage (ICH). *Patients and Methods*. The study included 75 patients with ICH of both sex and all age groups. ICH, based on CT scan findings, was divided in the following groups: lobar, subcortical, infratentorial, intraventricular haemorrhage and multiple hematomas. Volume of intracerebral hematoma was calculated according to formula *V* = 0.5 × *a* × *b* × *c*. Intracerebral hematomas, according to the volume, are divided in three groups (0–29 mL, 30–60 mL, and >60 mL). *Results*. The highest mortality rate was recorded in the group with multiple hematomas (41%), while the lowest in infratentorial (12.8%). The best six-month survival was in patients with a volume up to 29 mL, 30 of them (64%) survived. The highest mortality rate was recorded in patients with the hematoma volume >60 mL (85%). Kaplan-Meier's analysis showed that there was statistical significance between the size of the hematoma and the six-month survival (*P* < 0.0001). More than half of patients (61.1%) who survived 6 months after ICH were functionally independent (Rankin scale ≤2). *Conclusion* The volume of hematoma significantly affects six-month prognosis in patients with intracerebral hemorrhage, while localization does not.

## 1. Introduction

Intracerebral hemorrhage (ICH) is defined as bleeding into the parenchyma of the brain which may further extend into ventricles. ICH occurs in 15 to 20% of all strokes. Compared to ischemic stroke, it more often results in death and increased disability [1]. The most important risk factor is hypertension, which increases the risk of ICH by approximately 4 times. Improved hypertension control reduces the incidence of intracerebral haemorrhage [2]. The other causes of ICH are arteriovenous malformation, brain tumor, amyloid angiopathy, and blood or bleeding disorders. A three-month mortality of ICH is 34%, while only 31% of patients are functionally independent 3 months after ICH. ICH can be localized in the different parts of the brain (cerebral lobes, basal ganglia, thalamus, brainstem, and cerebellum), and large hematoma is accompanied with spreading of blood into ventricles [1].

The aim of this study was to determine whether the volume and localization of intracerebral hematoma affects the six-month prognosis of patients with ICH.

## 2. Patients and Methods

The study included 75 patients with ICH of both sexes and all age groups, hospitalized at the Department of Neurology, Tuzla, Bosnia and Herzegovina, in the period from June 2007 to March 2008. The inclusion criteria was ICH diagnosed on computed tomography, while the recurrence of ICH was the excluding criterion. All patients in our study were treated with conservative treatment. Localization of ICH, based on CT scan findings, was divided into the following groups: lobar, subcortical, infratentorial, intraventricular hemorrhage, and multiple hematomas. The volume of intracerebral hematoma was calculated according to the formula *V* = 0.5 × *a* × *b* × *c* (*a* is the largest diameter of hematoma, *b* the diameter versus *a*, and *c* the number of scans that shows hematoma) [3]. The thickness of the scan through the back of the skull was 4 mm and 6 mm through the other parts. When the first or the last scan recorded only a small amount of blood, the computer value *c* was calculated as 0.5 (half the thickness of the scan) and not as 1 (6, resp., 4 mm). Intracerebral hematomas are divided by the volume into three groups (0–29 mL, 30–60 mL, and >60 mL). Short-term outcome was defined as survival or death and functional dependence six months after ICH [4].

## 3. Statistical Analysis

SPSS 15.0 was used for statistical data analysis. Statistical parameters such as the mean and standard deviation were used in the analysis of results. We used chi-square (*χ* ^2^) test to assess the significance of differences, and we also used Kaplan-Meier's analysis of survival. A value of *P* < 0.05 was considered statistically significant. This study was approved by the Ethical Committee of the Tuzla University Clinical Centre.

## 4. Results

This study included 75 patients with ICH. The average age was 64.3 ± 13.7 (16–86), and men were significantly older (*P* = 0.031). Out of the total number of patients, 74 of them had spontaneous intracerebral hemorrhage, while only in one patient arteriovenous malformation was diagnosed as a cause of ICH. Six months after the onset of ICH, 36 (48%) patients survived. The highest mortality rate was in the group with multiple hematomas (41%), while the lowest was in infratentorial (12.8%). There was no statistical significance between the localization of the hematoma and the six-month outcome of patients (Table 1). 

**Figure 1:**
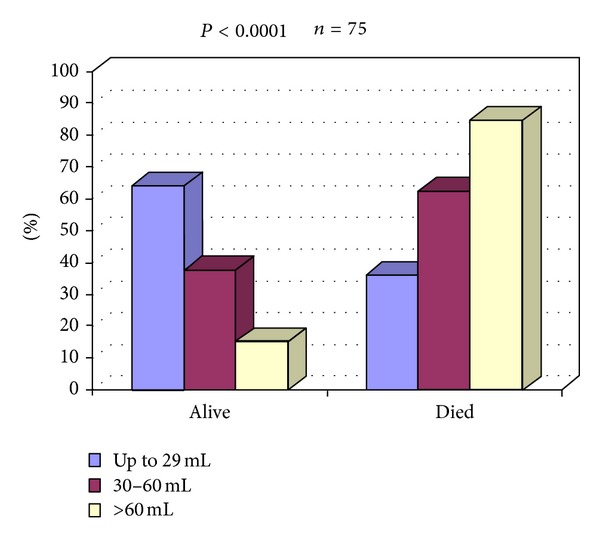
Six-month outcome of the patients with intracerebral hematoma according to the hematoma volume.

The best six-month survival was in patients with a volume up to 29 mL; 30 of them (64%) survived. The highest mortality rate was recorded in patients with the hematoma volume more than 60 mL (85%), followed by the group of 30 to 60 mL (62.5%), and the lowest mortality rate was among the patients with a hematoma volume up to 29 mL (36%) (Figure 1). 

**Figure 2:**
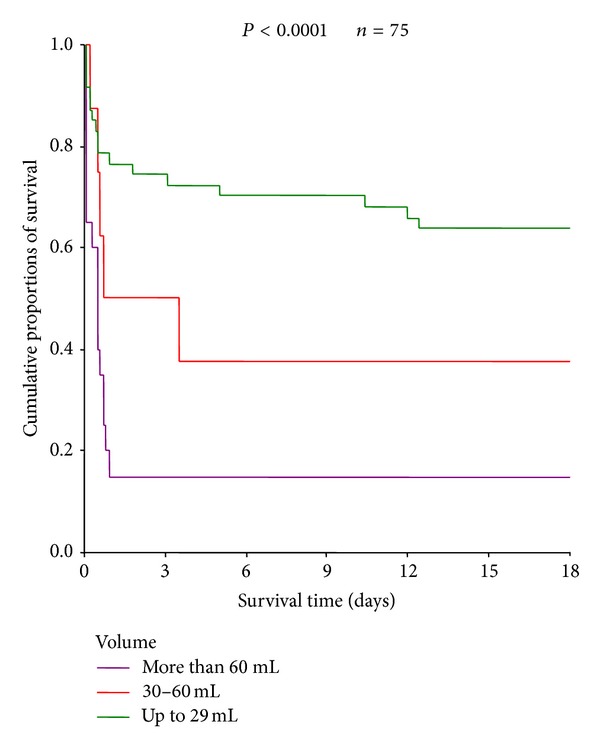
Survival of patients with intracerebral hemorrhage according to the hematoma volume.

Kaplan-Meier's analysis showed that there was a statistical significance between the volume of the hematoma and the six-month survival (*P* < 0.0001) (Figure 2).

More than half of patients (61.1%) who survived 6 months after stroke were functionally independent (Rankin scale ≤ 2) (Table 2). The volume of hematoma significantly influenced six-month prognosis (survival and functional dependency) in patients with ICH. 

## 5. Discussion

The results of short-term outcome regarding the localization of hematoma are varying from study to study. In many studies, the most favorable outcome was recorded in patients with subcortical hematomas, and the worst in the brainstem hematomas. In the study of Arboix et al. [5], the highest mortality rate was in patients with multiple hematomas, which is similar to our study. One Italian study [6] showed that the lowest mortality rate related to cerebellar hematomas, and the highest with hematomas located in pons. Their results are similar to the results of one of our earlier studies [7]. 

The volume of hematoma was an independent factor influencing mortality in patients with ICH [8]. Studies of Godoy et al. [6] and Togha and Bakhtavar [8] showed that unfavorable outcome (mortality) was higher in the groups with greater hematoma volume, which is similar to the results of our study. Castellanos et al. noted that the higher hematoma volume, deep localization, intraventricular spreading of hematoma, masseffect was significantly associated with a worse outcome [3]. Daverat et al. [9] and Lampl et al. [10] showed that hematoma volume influenced short-term prognosis patients with ICH, and it is similar with our study. In the recent study of Al-Khaled et al. [11], the mortality rate in ICH patients treated on conservative treatment was much lower in comparison with our study (18% versus 48%). 

In our study, more than half of patients were functionally independent (Rankin scale ≤ 2) six months after ICH. This is the result of the physical treatment conducted in different conditions (spa, clinical, or outpatients), and primarily it is the result of an early start of physical treatment, in the first days after the onset of ICH. Results of our study are better compared to the Italian study where only 40% of patients were functionally independent six months after ICH [9]. 

Finally, we can conclude that the volume of hematoma significantly affects six-month prognosis in patients with intracerebral hemorrhage, while localization does not.

## Figures and Tables

**Table 1:** Vital status of patients related to localization of hematoma six months after the onset of ICH.

Localization of hematoma	Alive (*N* = 36)		Died (*N* = 39)		Total (*N* = 75)		*P*
	*N*	%	*N*	%	*N*	%	
Lobar	12	33.3	11	28.2	23	30.7	0.8
Subcortical	11	30.5	7	17.9	18	24.0	0.2
Infratentorial	3	8.3	5	12.8	8	10.6	0.7
Multiple	8	22.2	16	41.0	24	32.0	0.09
IV hemorrhage	2	5.5	0	0.0	2	2.7	0.2

IV: intraventricular.

**Table 2:** Functionally dependency of patients with intracerebral hemorrhage compared to the hematoma volume.

Volume of hematoma	Rankin scale ≤2		Rankin scale >2	
	*N*	%	*N*	%
Up to 29 mL	21	58.3	10	27.7
30–60 mL	1	2.7	2	5.6
>60 mL	0	0.0	2	5.6

Total	22	61.1	14	38.9
